# Comparative Study of Somatostatin-Human Serum Albumin Fusion Proteins and Natural Somatostatin on Receptor Binding, Internalization and Activation

**DOI:** 10.1371/journal.pone.0089932

**Published:** 2014-02-27

**Authors:** Ying Peng, Lili Deng, Yuedi Ding, Quancheng Chen, Yu Wu, Meilin Yang, Yaping Wang, Qiang Fu

**Affiliations:** 1 Key Laboratory of Nuclear Medicine, Ministry of Health, Jiangsu Key Laboratory of Molecular Nuclear Medicine, Jiangsu Institute of Nuclear Medicine, Wuxi, Jiangsu, China; 2 The First Affiliated Hospital of Nanjing Medical University, Nanjing, Jiangsu, China; 3 Wuxi Second People’s Hospital of Nanjing Medical University, Wuxi, Jiangsu, China; Medical School of Hannover, Germany

## Abstract

Albumin fusion technology, the combination of small molecular proteins or peptides with human serum albumin (HSA), is an effective method for improving the medicinal values of natural small molecular proteins or peptides. However, comparative studies between HSA-fusion proteins or peptides and the parent small molecules in biological and molecular mechanisms are less reported. In this study, we examined the binding property of two novel somatostatin-HSA fusion proteins, (SST14)_2_-HSA and (SST28)_2_-HSA, to human SSTRs in stably expressing SSTR1-5 HEK 293 cells; observed the regulation of receptor internalization and internalized receptor recycling; and detected the receptors activation of HSA fusion proteins in stably expressing SSTR2- and SSTR3-EGFP cells. We showed that both somatostatin-HSA fusion proteins had high affinity to all five SSTRs, stimulated the ERK1/2 phosphorylation and persistently inhibited the accumulation of forskolin-stimulated cAMP in SSTR2- and SSTR3-expressing cells; but were less potent than the synthetic somatostatin-14 (SST-14). Our experiments also showed that somatostatin-HSA fusion proteins did not induce the receptors internalization; rather, they accelerated the recycling of the internalized receptors induced by SST-14 to the plasma membrane. Our results indicated that somatostatin-HSA fusion proteins, different from SST-14, exhibit some particular properties in binding, regulating, and activating somatostatin receptors.

## Introduction

Somatostatin (SST) is a secretory product of a variety of endocrine and non-endocrine tissues and is widely distributed throughout the body [1], [2]. Somatostatin inhibits pituitary, pancreatic, and gastrointestinal hormone secretion release, as well as cytokines production, intestinal motility and absorption, vascular contractility, and cell proliferation [2]. The somatostatin molecule has two biologically active forms: somatostatin-14 (SST-14), the cyclic tetradecapeptide, and somatostatin-28 (SST-28), an N-terminally elongated form of SST-14 [1]. Both forms of somatostatin exert biological function through receptors on target cells and intracellular pathways. Five subtypes of somatostatin receptors (SSTR 1–5) have been recognized, with two spliced variants for SSTR2: SSTR2A and SSTR2B, with a different carboxyl terminus [3].

The beneficial effects of somatostatin in the treatment of some hypersecretory disorders of hormone excess and its antiproliferation effect on tumors are well recognized [4], [5]. However, the clinical utility of natural somatostatin is limited due to 2–3 min half-life in vivo due to enzymatic degradation and endocytosis [6], [7]. In the past decade, numerous biologically stable somatostatin analogs have been developed by a variety of molecular modifications of natural somatostatin. Some of them, for example octreotide and lanreotide, are being clinically used in treatment of GH-secreting adenomas and carcinoids, but therapeutic limitations still exist due to altered binding affinity to SSTRs [8].

Albumin, the most abundant protein in the blood plasma, is produced in the liver as a monomeric protein of 67 kDa and responsible for 80% of the colloid osmotic pressure of plasma [9]. Albumin fusion technology markedly prolongs proteins or peptides half-life in vivo by combining the target proteins or peptides with human serum albumin (HSA) and improves the therapeutic activity of target proteins, due to the reduction of renal clearance and the metabolicapp:addword:metabolic stability of HSA [10]. For examples, human granulocyte colony stimulating factor (G-CSF) [11], human growth hormone (GH) [12], human insulin [13], human interferon-α-2b (INF-2b) [14], and interleukin-28B (IL-28B) [15] fused with HSA were used effectively to construct long-acting therapeutic drug candidates. However, the comparative studies between HSA fusion proteins and the parent molecules in the biological and molecular mechanisms are less reported [13], [16].

Here, we side-by-side compared the binding affinity of two novel genetic fusion products (SST14)_2_-HSA and (SST28)_2_-HSA, which were constructed by fusing two copies of SST-14 or SST-28 at N-terminus of HSA, with human SSTRs and observed the effects of fusion proteins on receptor internalization, recycling and activation in HEK 293 cells which stably expressing human somatostatin receptor subtype.

## Materials and Methods

### Reagents and Antibodies

Fetal bovine serum (FBS), L-glutamine, antibiotics, Dulbecco’s modified Eagle’s medium (DMEM) and trypsin/EDTA were obtained from Gibco (Life Technologies, Grand Island, NY, USA). Synthetic somatostatin-14 (S1763) and [Tyr^1^]-somatostatin (S4633), 1-methyl-3-isobutylxanthine (I5879) and forskolin (F6886) were purchased from Sigma (Sigma-Aldrich, St. Louis, MO, USA). Anti-SSTR3 (ab137026) antibody was purchased from Abcam (Abcam, London, UK). Anti-SSTR1 (C-17), anti-SSTR2 (H-50), anti-SSTR4 (H-50), anti-SSTR5 (H-54), anti-p-ERK (E-4), anti-ERK1 (K-23), anti-β-Actin (ACTBD11B7) antibodies and HRP-conjugated secondary antibody were purchased from Santa Cruz (Santa Cruz Biotechnology, Inc., Santa Cruz, CA, USA).

### Fusion Proteins Production

(SST14)_2_-HSA and (SST28)_2_-HSA fusion proteins were generated by *Pichia pastoris* expression system in our lab as previously described [17] and stored frozen at −80°C. The HSA gene (NM_000477) was amplified from the pBluescript II KS (+)-hsa and subcloned into the pMD19-T vector. Two copies of somatostatin-14 [(SST14)_2_] with nuclear sequences of 5′-gct ggc tgc aag aat ttc ttc tgg aag act ttc aca tcc tgt gct ggc tgc aag aat ttc ttc tgg aag act ttc aca tcc tgt-3′; or somatostatin-28 [(SST28)_2_] with nuclear sequences of 5′-tct gct aac tca aac ccg gct atg gca ccc cga gaa cgc aaa gct ggc tgc aag aat ttc ttc tgg aag act ttc aca tcc tgt tct gct aac tca aac ccg gct atg gca ccc cga gaa cgc aaa gct ggc tgc aag aat ttc ttc tgg aag act ttc aca tcc tgt-3′ were obtained by overlapping PCR and subcloned into pMD19T-hsa vector. Finally, the constructed fusion genes of (SST14)_2_-HSA or (SST28)_2_-HSA were digested with EcoR I and Not I and then inserted into the corresponding sites of the *P. pastoris* expression vector pPIC9K.

### DNA Construction

Human SSTR1-5 cDNA sequences containing the ORF from SSTR1 (GenBank NM_001049), SSTR2 (GenBank NM_001050), SSTR3 (GenBank NM_001051), SSTR4 (GenBank NM_001052) and SSTR5 (GenBank NM_001053) were PCR amplified from reverse transcribed cDNA of human small intestinal biopsy tissues and subcloned into the EGFP removed pEGFP-N1 (Clontech) vector via TOPO cloning (Invitrogen, Carlsbad, CA). SSTR2- and SSTR3-EGFP vectors were generated by respectively inserting SSTR2 and SSTR3 ORF sequence into Xho I and Hind III sites of pEGFP-N1. All clones were verified by sequencing carried out by Sangon Biotech Co. Ltd. (Shanghai, China).

### Cell Culture and Transfection

Human embryonic kidney (HEK) 293 cells (bought from ATCC) were grown in Dulbecco’s modified Eagle’s medium (DMEM) supplemented with 10%(v/v) heat inactivated fetal calf serum, 100 units/ml penicillin, 100 µg/ml streptomycin in a humidified atmosphere containing 5% CO_2_. Transfections were performed using Lipofectamine 2000 (Invitrogen) according to the instructions of the manufacturer. Stably transfected cells were selected in the presence of 400 µg/ml G418 (Sigma) and then maintained in the presence of 200 µg/ml G418. Pools of stably transfected cells for each construct were used for this study.

### Identification of HEK 293 Cells Stably Expressing Human SSTR1-5

HEK 293 cells stably expressing SSTR1-5, or SSTR2- and SSTR3-EGFP were characterized using reverse transcription polymerase chain reaction (RT-PCR), western blot analysis and fluorescence microscope. The mRNA extracted from stable expressing SSTR1-5 HEK 293 cells were first reverse transcribed to cDNA with High Capacity cDNA Reverse Transcription Kit (Applied biosystems, Carlsbad, CA, USA). The PCR was performed with primers listed in Table S1. The PCR products were electrophoresed through a 1% agarose gel. For western blot analysis, total membrane protein was isolated by ProteoExtract Transmembrane Protein Extraction Kit (Novagen) and separated with a 10% SDS-PAGE gel. After blotting to PVDF membrane (Life Technologies, Grand Island, NY, USA), membranes were blocked for 60 minutes at room temperature and incubated overnight at 4°C in buffer (TBS with 0.1% Tween 20 and 5% non-fat milk power) containing 1∶500 diluted somatostatin antibodies. Detection of primary antibodies was performed with HRP-conjugated secondary antibody. Immunoreactive bands were visualized with ECL western blotting luminol reagent (Santa Cruz).

### Localization and Observation of Receptors in Live Cells

HEK 293 cells stably expressing SSTR2- and SSTR3-EGFP were stained with LysoTracker Red DND-99 (Invitrogen) according to the manufacturers’ protocols to observe subcellular localization. Receptor internalization and recycling were detected using the Olympus IX51 fluorescence microscope.

### Whole Cell Binding Assays

Binding experiments of (SST14)_2_-HSA and (SST28)_2_-HSA for SSTRs were performed with HEK 293 cells stably expressing SSTR1-5 respectively. Labeling of Tyr^1^-somatostatin with ^125^I was performed using the chloramine-T method [18]. PD-10 Sephadex G-25M column (GE Healthcare) were used for further purification of the labeled tracer. HEK 293 cells stably expressing SSTR1-5 were seeded in 24-well plates at a density of 1.5×10^5^/well. 12 hours later, the growth medium was replaced with 0.2% bovine serum albumin in DMEM and cells were continuously incubated for another 2 hours at 37°C. Then, the medium was aspirated, and 0.25 ml of binding medium containing 50 pM ^125^I-Tyr^1^-somatostatin alone or with increasing concentrations of unlabeled (SST14)_2_-HSA or (SST28)_2_-HSA was added to the cells and incubated at 25°C for 1 hour. After that, the cells were washed twice with 0.5 ml of ice-cold phosphate-buffered saline to remove nonspecifically bound tracer. 0.5 ml of 1 N NaOH was added to each well and incubated at room temperature to solubilize the cells. Radioactivity was measured in a Perkin Elmer 1470 automatic γ counter [19], [20].

### ERK Assays

The cells were seeded onto 6-well dishes at a density of 3.5×10^5^/well and grown for 48 hours in DMEM containing 0.5% fetal calf serum. The cells were then exposed to SST-14, (SST14)_2_-HSA, or (SST28)_2_-HSA in different concentrations in serum-free DMEM medium at 37°C for 5 minutes. The reaction was terminated by removal of the culture medium and addition of 900 µl boiling 1×SDS sample buffer. Samples were collected and heated at 95°C for an additional 5 minutes period [8]. Equal amounts of protein of each sample were separated with 10% SDS-PAGE gels and transferred to PVDF membranes. After blocking, the membranes were incubated with 1∶1000 diluted anti-p-ERK or anti-ERK1/2 antibodies overnight at 4°C. Blots were detected using HRP-conjugated secondary antibody and the ECL western blotting luminol reagent. Densitometric analysis was performed using ImageJ software.

### Measurements of cAMP Accumulation

Transfected cells were seeded in 24-well plates at a density of 1.0×10^5^/well. The next day, the cells were washed and incubated with 0.25 ml of serum-free DMEM medium containing 0.75 mM 1-methyl-3-isobutylxanthine and 25 µM forskolin or 25 µM forskolin plus SST-14, (SST14)_2_-HSA, or (SST28)_2_-HSA in concentrations ranging from 10^−12^ to 10^−5^ M. The cells were incubated at 37°C for 15 minutes then the reaction was terminated by removal of the culture medium and addition of 0.2 ml of 0.1 M HCl with 0.1% Triton X-100 (Sigma). The cAMP content was measured using a commercial cAMP ELISA kit (NewEast Biosciences; Malvern, PA, USA) following the manufacturers’ protocols.

### Data Analysis

Results were expressed as the mean ± S.E. Statistical significance was identified by one-way anova, with probability *p*<0.05 being considered significant. The data were graphed using GraphPad Prism 5.0 software.

## Results

### Binding Affinity of Somatostatin-HSA Fusion Proteins for Somatostatin Receptors

**Figure 1:**
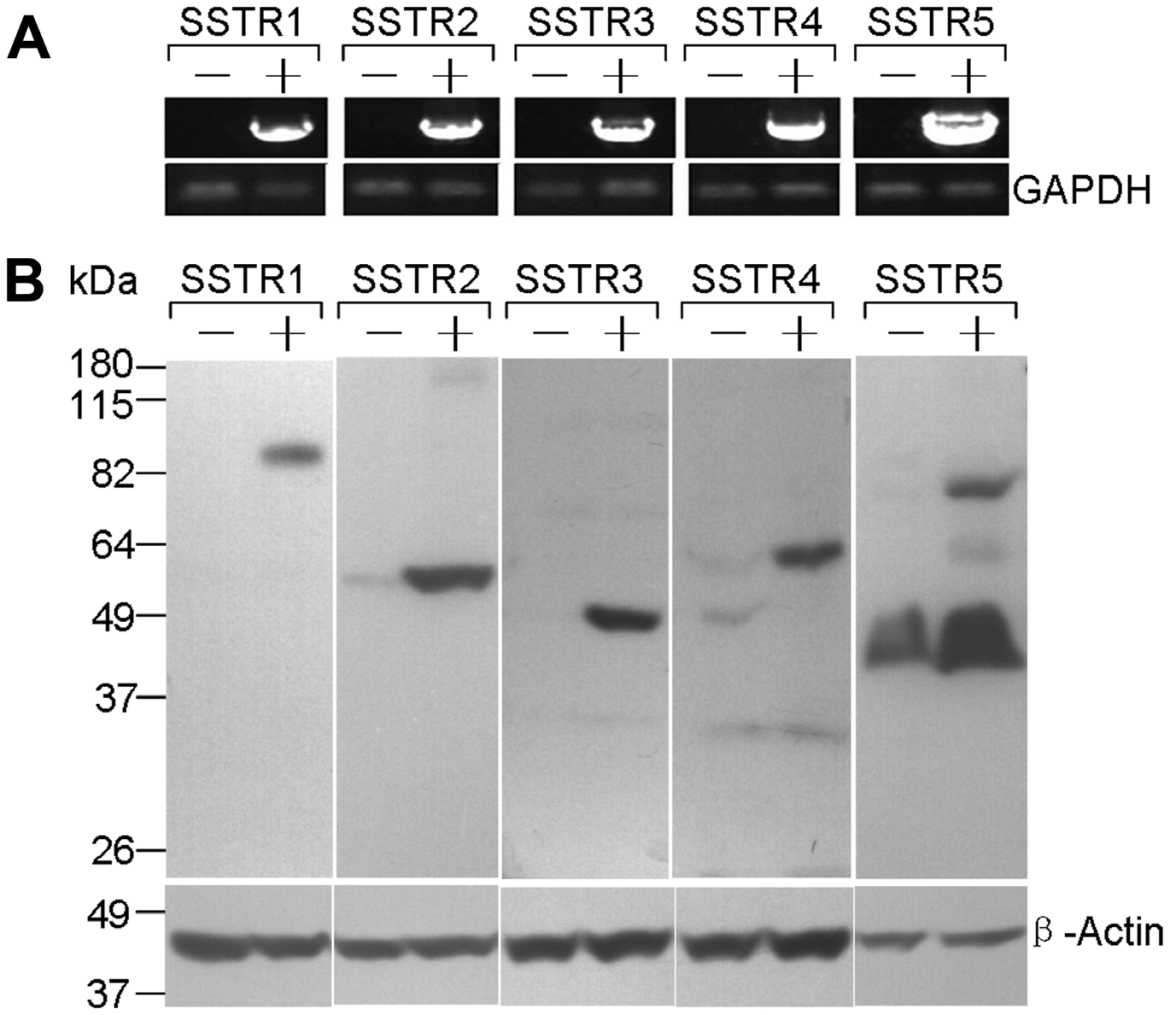
Identification of HEK 293 cells stably expressing human SSTR1-5. (A) RT-PCR analysis showing mRNA expression of SSTR1-5 in either HEK 293 cells or HEK 293 cells transfected with SSTR1-5 respectively. 1.5 µg of DNA-free RNA was reverse transcribed and co-amplified with primers specific for SSTR1-5 and GAPDH. 5 µL of PCR products and fractionated on agarose gels then visualized under UV lighting. (B) Western blot analysis showing protein expression of SSTR1-5 in either HEK 293 cells or HEK 293 cells transfected with SSTR1-5 respectively. 25 µg of protein was fractionated by SDS-PAGE and probed with appropriate SSTR antibodies.

**Figure 2:**
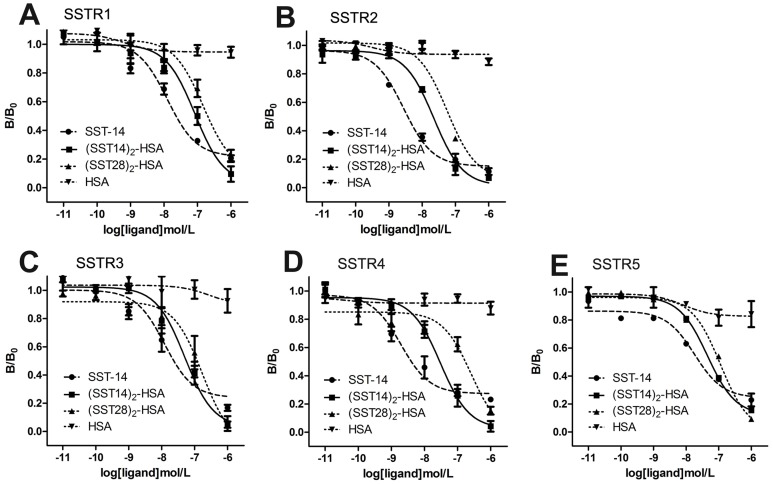
Binding affinity of somatstatin-HSA fusion proteins for somatostatin receptors. HEK 293 cells stably expressing SSTR1, SSTR2, SSTR3, SSTR4 or SSTR5 were incubated with 50^125^I-Tyr^1^-somatostatin in the absence or presence of increasing concentrations of unlabeled ligand for 1 hour at 25°C. After washing to remove nonspecific binding, the amount of bound radioligand was quantitated in a γ-counter. Data are presented as the mean of three independent experiments. S.E. values were smaller than 15%.

SST-14 has universal high-affinity binding to all somatostatin receptor subtypes SSTR1-5 [21]. To investigate the binding property of (SST14)_2_-HSA and (SST28)_2_-HSA with SSTRs, we generated HEK 293 cells which stably expressed each individual human somatostatin receptor subtype. The mRNA and protein expression of SSTR1-5 were identified by RT-PCR and western blot analysis, as shown in Figure 1A and B. The competitive ^125^I-Tyr^1^-somatostatin binding experiments were performed to determine the binding affinity of fusion proteins to SSTRs on whole HEK 293 cells respectively expressing SSTR1-5. The results demonstrated that both fusion proteins had high affinity to all of five receptors with half-maximal inhibitory concentrations (IC50) in nanomolar range. Relative to (SST14)_2_-HSA, (SST28)_2_-HSA had lower binding affinities to SSTRs and the *Pichia pastoris* expressed HSA had no significant binding with SSTRs. In addition, (SST14)_2_-HSA and (SST28)_2_-HSA exhibited the different binding profile for each receptor subtype. (SST14)_2_-HSA had high affinity to SSTR2, moderate affinity to SSTR3, SSTR4 and SSTR5, and low affinity to SSTR1, but (SST28)_2_-HSA only had high affinity to SSTR2 and low affinity to other receptor subtypes (Figure 2A-E and Table 1).

**Table 1:** Ligand binding properties of (SST14)_2_-HSA and (SST28)_2_-HAS.

	Ligand binding affinity IC_50_ (nM)				
Ligand	SSTR1	SSTR2	SSTR3	SSTR4	SSTR5
SST-14	12.54	2.77	12.21	1.93	18.21
(SST14)_2_-HSA	84.51	22.43	50.22	30.39	45.19
(SST28)_2_-HSA	147.91	56.82	172.11	257.02	114.12

Radioligand binding studies were carried out as described under “Materials and Methods”. The half-maximal inhibitory concentrations (IC50) for competition binding assays were analyzed by nonlinear regression curve fitting using the computer program GraphPad Prism 5.0. Data are presented as the mean of three independent experiments. S.E. values were smaller than 15%.

### Regulation of Somatostatin-HSA Fusion Proteins to Receptors Internalization and Recycling in SSTR2- and SSTR3-expressing Cells

**Figure 3:**
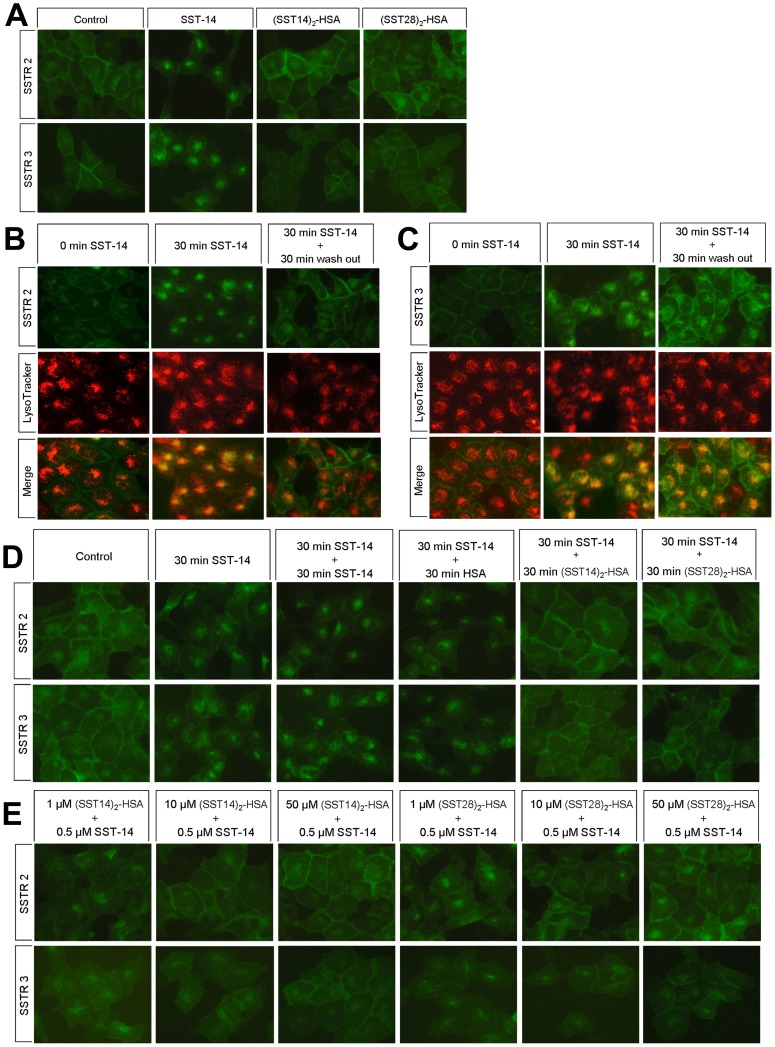
Regulation of somatostatin-HSA fusion proteins to receptors internalization and recycling in SSTR2- and SSTR3-expressing cells. (A) Comparison of agonist-induced endocytosis of SSTR2 and SSTR3. HEK 293 cells stably expressing SSTR2- or SSTR3-EGFP were exposed to 1 µM SST-14, (SST14)_2_-HSA or (SST28)_2_-HSA for 0 or 30 minutes. The subcellular distribution of receptor protein was examined by fluorescence microscopy. (B, C) Internalization and recycling of SSTR2 and SSTR3. HEK 293 cells expressing SSTR2- or SSTR3-EGFP were exposed to 0.5 µM SST-14 for 30 minutes. Cells were then washed and followed by an agonist-free interval of 30 minutes. Cells were stained with LysoTracker Red DND-99 according to the manufacturers’ protocols. Locations of SSTR2 or SSTR3 (upper panel, green) and lysosomes (middle panel, red) were examined by fluorescence microscopy. The merged image (lower panel) shows co-localization (yellow) of SSTR2 or SSTR3 and lysosomes. Images were processed using ImageJ software. (D) Fusion proteins accelerated the recycling of internalized receptors to the plasma membrane. SSTR2- or SSTR3-EGFP-expressing HEK 293 cells were treated by 0.5 µM SST-14 for 30 minutes, then 50 µM of SST-14, HSA, (SST14)_2_-HSA or (SST28)_2_-HSA were added with little change in final volume. After 30 minutes treatment, the subcellular distribution of receptor protein was examined by fluorescence microscope. (E) Dose-dependent inhibition of receptors internalization. SSTR2- or SSTR3-EGFP-expressing HEK 293 cells were exposed to 1 µM, 10 µM or 50 µM (SST14)_2_-HSA or (SST28)_2_-HSA for 30 minutes, then 0.5 µM SST-14 were added while final volume changed little. After 30 minutes treatment, the subcellular distribution of receptor was examined by fluorescence microscope. Representative results from one of three independent experiments performed in duplicate are shown.

Receptor internalization is one of main processes that regulate the activity of SSTRs [22]. To observe the regulation of fusion proteins for somatostatin receptors, we selectively generated cells that stably expressed EGFP-tagged SSTR2 and SSTR3 subtype. The EGFP protein was inserted to C-terminal of SSTR2 and SSTR3, which allowed direct observation of the subcellular localization of somatostatin receptor after fusion proteins treatments. Similar to previous reports [8], [23], in untreated cells, SSTR2 and SSTR3 were almost exclusively confined to the plasma membrane and SST-14 induced rapidly internalization of both receptor subtypes (Figure 3A); the internalized SSTR2 and SSTR3 induced by SST-14 rapidly recycled to the plasma membrane after being washed extensively and subjected to an additional incubation in the absence of SST-14 for 30 minutes, as depicted in Figure 3B, C. However, (SST14)_2_-HSA and (SST28)_2_-HSA fusion proteins did not obviously induce receptor internalization as observed by fluorescence microscope (Figure 3A). Both fusion proteins accelerated the recycling of internalized receptors to the plasma membrane. HSA alone did not have any effects on the recycle of SSTRs (Figure 3D). In addition, cells were pre-treated with (SST14)_2_-HSA or (SST28)_2_-HSA for 30 min, both fusion proteins dose-dependently blocked receptor subtypes internalization induced by SST-14 (Figure 3E).

### Phosphorylation of ERKs Induced by Somatostatin-HSA Fusion Proteins in SSTR2- and SSTR3-expressing Cells

**Figure 4:**
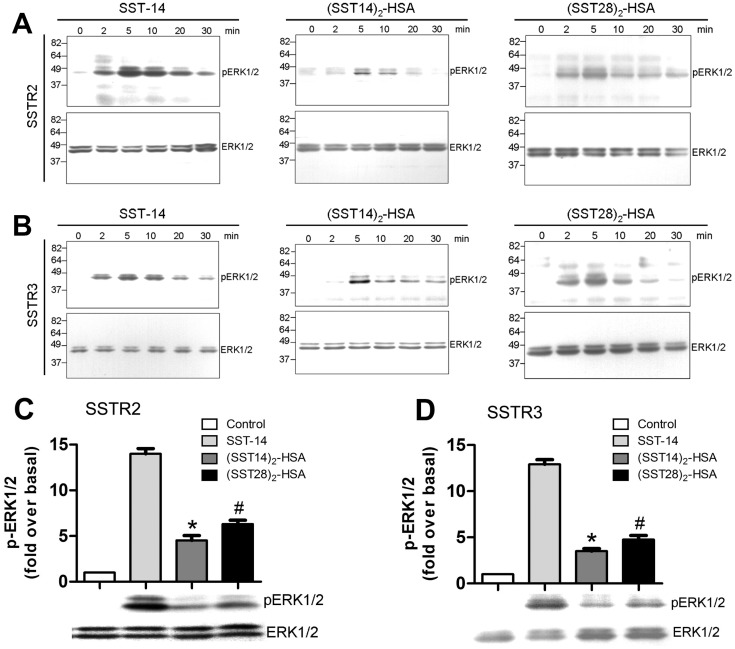
Comparison of SST-14-, (SST14)_2_-HSA- and (SST28)_2_-HSA-induced ERK activation in SSTR2- and SSTR3-expressing cells. (A, B) Time-dependent ERK1/2 activation. HEK 293 cells expressing SSTR2 or SSTR3 were exposed to 1 µM SST-14, (SST14)_2_-HSA, or (SST28)_2_-HSA for 0, 2, 5, 10, 20, or 30 minutes. Cells were lysed, equal amounts of protein were resolved by SDS-PAGE, and levels of total ERK1/2 and phosphorylated ERK1/2 were determined by immunoblotting. Two additional experiments gave similar results. (C, D) Agonist-induced ERK1/2 activation. HEK 293 cells expressing SSTR2 or SSTR3 were exposed to 1 µM SST-14, (SST14)_2_-HSA or (SST28)_2_-HSA for 5 minutes. The cells were lysed, equal amounts of protein were resolved by SDS-PAGE, and the levels of total ERK1/2 and phosphorylated ERK1/2 were determined by immunoblotting. Results were quantified by densitometric analysis. The data were normalized to total ERK1/2 and expressed as the fold ERK1/2 phosphorylation over the basal value in untreated cells. The values represent the means ± S.E. of three independent experiments performed in duplicate. *, *p*<0.01, (SST14)_2_-HSA-treated cells compared with SST-14-treated cells. #, *p*<0.01, (SST28)_2_-HSA-treated cells compared with SST-14-treated cells.

The MAP kinase ERK1/2 is the most important downstream signaling molecule that is involved in mediating SST-14 inhibition of cell proliferation [24]. To explore the receptor activation after fusion protein treatment, we investigated the ability of fusion proteins to increase the levels of phosphorylated ERK1/2 in SSTR2- and SSTR3-expressing cells. As shown in Figure 4A, B, similar to SST-14, exposure to (SST14)_2_-HSA or (SST28)_2_-HSA led to a rapid and transient phosphoryation of ERK1/2 in SSTR2- and SSTR3-expressing cells with a maximum time of around 5 minutes, but less potency than SST-14 in same molecular concentration (Figure 4C, D). Compared with (SST14)_2_-HSA, (SST28)_2_-HSA had more potency for induction of ERK1/2 phosphorylation (Figure 4C, D).

### Inhibition of Somatostatin-HSA Fusion Proteins to cAMP Accumulation in SSTR2- and SSTR3-expressing Cells

**Figure 5:**
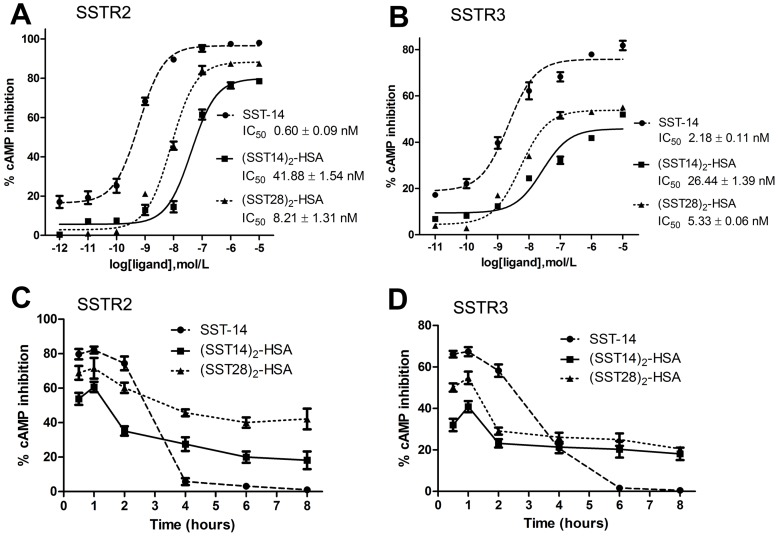
Comparison of SST-14-, (SST14)_2_-HSA- and (SST28)_2_-HSA-induced adenylyl cyclase inhibition in SSTR2- and SSTR3-expressing cells. (A, B) Dose-dependent inhibition for forskolin-stimulated cAMP accumulation. HEK 293 cells expressing SSTR2 or SSTR3 were exposed to 25 µM forskolin plus SST-14, (SST14)_2_-HSA or (SST28)_2_-HSA in concentrations ranging from 10^−12^ to 10^−5^ M for 15 minutes. Cells were lysed and cAMP levels were determined using a cAMP ELISA kit. The maximum forskolin-stimulated cAMP formation in the absence of agonist was defined as 100%. Values represent the means ± S.E. of three separate measurements performed in triplicate. The half-maximal inhibitory concentrations (IC50) were analyzed by nonlinear regression curve fitting using GraphPad Prism 5.0. (C, D) Time-dependent inhibition for forskolin-stimulated cAMP accumulation. HEK 293 cells expressing SSTR2 or SSTR3 were pre-treated with 1 µM SST-14, (SST14)_2_-HSA or (SST28)_2_-HSA, at 0.5, 1, 2, 4, 6, 8 hour time points, cells were exposed to 25 µM forskolin for 15 minutes. Cells were lysed and cAMP levels were determined using a cAMP ELISA kit. The maximum forskolin-stimulated cAMP formation in the absence of agonist was defined as 100%. Values represent the means ± S.E. of three separate measurements performed in triplicate.

All somatostatin receptors are coupled to inhibitory G proteins, thereby mediating inhibition of cAMP accumulation [25]. To further investigate the receptor activation of fusion proteins, the ability of fusion proteins to inhibit forskolin-stimulated cAMP accumulation in cells expressing SSTR2 and SSTR3 was measured. The results shown that both fusion proteins dose-dependently inhibited intracellular cAMP accumulation. Compared to SST-14, the IC50 was 70- or 10-fold higher in SSTR2-expressing cells for (SST14)_2_-HSA or (SST28)_2_-HSA (Figure 5A), or 10- and 2-fold higher in SSTR3-expressing cells respectively (Figure 5B), however, both fusion proteins had persistent effects for forskolin-stimulated cAMP accumulation in SSTR2- and SSTR3-expressing cells. Eight hours after one-time fusion proteins exposure, the forskolin-stimulated cAMP accumulation in SSTR2- and SSTR3-expressing cells was still 20% inhibited. In contrast, the inhibition of SST-14 for forskolin-stimulated cAMP accumulation was completely eliminated at four hours in SSTR2-expressing cells or at six hours in SSTR3-expressing cells after exposure (Figure 5C, D).

## Discussion

In this study, we compared the binding property to receptors, the regulation of internalization and internalized receptor recycling, as well as the capability to activate receptors between somatostatin-HSA fusion proteins and SST-14. Our results suggested that there are some differences in molecular mechanisms between somatostatin fusion proteins and SST-14. We showed that although the binding affinity is lower than SST-14, somatostatin fusion proteins bind to all of five somatostatin receptors and maintain a long-term receptor signaling. In addition, the fusion proteins do not trigger, but instead interfere with the receptors internalization, and accelerate the recycling of internalized receptors to the plasma membrane.

Compared to their parent molecules, the lower biological potency in vitro assay had shown in most small molecular proteins- or peptides-HSA fusion products reported [26], [27]. It was speculated that the increase in molecular size after small parent molecules fused with albumin possibly reduced the numbers of receptors occupied and changed the rates of receptor dissociation and association. In our experimental system, the receptor affinities and activation of somatostatin-HSA fusion proteins were approximately 10 to 20 times less potent than SST-14, however, the fusion proteins had more long-term biological activation as measured by ERKs phosphoryation and cAMP accumulation.

SSTR2 is widely and strongly expressed in nervous, endocrine, and tumor tissues, and most of the clinically available somatostatin analogs have a strong SSTR2-binding affinity [28], [29]. SSTR3 uniquely triggers phosphotyrosine phosphatase-dependent apoptosis accompanied by activation of p53 and the pro-apoptotic protein Bax [30]. The internalization study of SSTR2 and SSTR3 after somatostatin analog exposure indicated that SSTR2 was rapidly recycled to the plasma membrane, whereas SSTR3 was subject to ubiquitination-dependent lysosomal degradation [23]. To investigate the regulation of fusion proteins for somatostatin receptors, we selectively compared the capability of fusion proteins with SST-14 to induce somatostatin receptor internalization by direct observation via fluorescence microscopy in SSTR2- or SSTR3-EGFP cell lines. Different from SST-14, in our experiment, we found that both somatostatin-HSA fusion proteins did not obviously trigger receptors internalization, and the internalization of SST-14 induced was dose-dependently inhibited by pre-treated with both fusion proteins. Furthermore, fusions proteins significantly facilitated the rapid recycling of internalized receptors to plasma membrane. A possible explanation for our finding is the ligand-induced conformation change was interfered as the dramatic increase of molecular mass and the change of molecular space structure after SST-14 or SST-28 fused with HSA, or the albumin domain of fusion protein may influenced the internalization of somatostatin receptor. Receptor internalization depends on the posttranslational modification of receptor through phosphorylation of specific residues. Researches had shown that the internalization of somatostatin receptors involved in the ligand-induced conformational change of receptors, receptor phosphorylation by G protein-coupled receptor kinase2 (GRK2), GRK3, PKC or some other kinase, as well as recruitment of β-arrestins [31]–[33]. The endocytosis of somatostatin analogue was significantly decreased by albumin [34].

It had been reported that majority of internalized SSTR3 is degraded in lysosomes, with only a small amount of internalized receptor being recycled to the cell surface [8], [23]. We observed the SSTR3, located at the plasma membrane, being internalized and then located in lysosomes after 30 minutes treatment with SST-14, as shown by living cells stained with LysoTracker Red DND-99. However, our results show that most of the internalized SSTR3 was recycled to plasma membrane after withdrawal of SST-14 for 30 minutes. In this study, the internalization and trafficking of SSTR3 was directly observed by fluorescence microscope in SSTR3-EGFP stably expressing HEK 293 cells, whereas the endocytosis or internalization of SSTRs in most of the previous reports were detected via an immunocytochemical method. It is not clear whether the discrepancy between our observation and previous reports is due to the difference in the experimental method or the existence of another trafficking pathway for SSTR3 and other receptors. Recent reports have shown that membrane localized receptors can also be stored in secretory lysosomes and transported from lysosome to plasma membrane in a manner dependent on stimulation [35], [36]. Whether this mechanism is also involved in SSTR3 or other somatostatin receptors trafficking is unknown, it remains to be determined in further studies.

In conclusion, our results suggested that small molecular-HSA fusion proteins exhibit some particular properties in binding, regulating, and activating their receptors. Better understanding of the molecular mechanisms is important for designing drugs using fusion proteins and anticipating their therapeutic effects, therefore, further research in this area is needed.

## Supporting Information

Table S1 **Primer sequences used in the identification of HEK 293 cells stably expressing human SSTR1-5.**
(DOC)
