# Supplementary material for: Comparative Study of Somatostatin-Human Serum Albumin Fusion Proteins and Natural Somatostatin on Receptor Binding, Internalization and Activation
Source: PLoS One. 2014 Feb 27;9(2):e89932. doi: 10.1371/journal.pone.0089932 (PMC3937410; doi:10.1371/journal.pone.0089932)
Supplement: Table S1 — Primer sequences used in the identification of HEK 293 cells stably expressing human SSTR1-5. (DOC) [file pone.0089932.s001.doc]

**Table S1. Primer sequences used in the identification of HEK 293 cells stably expressing human SSTR1-5**

| Primers | Nucleotide sequence (5´ to 3´ ) |
| --- | --- |
| SSTR1-F | AGCCTCGAGATGTTCCCCAATGGCACC |
| SSTR1-R | GCCAAGCTTGGCGAGCGTCGTGATCC |
| SSTR2-F | AGCCTCGAGATGGACATGGCGGATGAG |
| SSTR2-R | GCCAAGCTTGGCGATACTGGTTTGGAGG |
| SSTR3-F | AGCCTCGAGATGGACATGCTTCATCC |
| SSTR3-R | GCCAAGCTTGGCCAGGTAGCTGATG |
| SSTR4-F | AGCCTCGAGATGAGCGCCCCCTCGAC |
| SSTR4-R | GCCAAGCTTGGCGAAGGTGGTGGTCCTG |
| SSTR5-F | AGCCTCGAGATGGAGCCCCTGTTCCCAGC |
| SSTR5-R | GCCAAGCTTGGCCAGCTTGCTGGTCTGC |
